# Perfused human organs versus Mary Shelley's Frankenstein

**DOI:** 10.1186/1479-5876-7-9

**Published:** 2009-01-23

**Authors:** Lawrence Leung

**Affiliations:** 1 Department of Family Medicine, Queen's University, 220 Bagot Street, PO Bag 8888, Kingston Ontario K7L 5E9, Canada

## Abstract

Novel drugs have to go through mandatory pre-clinical testing before they can be approved for use in clinical trials. In essence, it is a form of bench-to-bedside (N2B) translational medicine, but the wastage rate of target candidates is immensely high. Effects seen *in vitro *often do not translate to *in vivo *human settings. The search is on for better models closer to human physiology to be used in pre-clinical drug screening. The Ex Vivo Metrics^© ^system has been introduced where a human organ is harvested and revitalized in a controlled environment suitable for testing of both drug efficacy and potential toxicity. This commentary expresses the author's views regarding this technology of perfused human organs.

## Introduction

Every new drug has to undergo Phase 1/2 clinical trials, where the safety and toxicity profile have to be clearly established before it can proceed to larger scale Phase 3/4 trials. Before entering any clinical phase, pre-clinical data would have to be procured from cultured cell-lines and tissues, in addition to animal models in the laboratories. However, no matter how good these models are, pre-clinical data may not be directly conversant with the natural physiology and processes of living human beings. This accounts for the immense attrition rate of research products from pharmaceutical research and development to clinical studies, and the initial setbacks of gene therapy when performed in humans[1], although some success has been seen more recently in gene therapy for selected diseases, for example, Parkinson's disease or lymphoid immunodeficiencies[2,3].

In line with N2B (Bench-to-Bedside) translational medicine philosophy, the search continues for a modeling system that operates in a way that resembles the human body as closely as possible, hence the advent of using perfused human organs. As exemplified by the Ex Vivo Metrics^©^[4], a human organ can be harvested and revitalized with matched blood in a control-simulated environment. The trial drug would be given via appropriate routes, and subsequent biochemical analysis would be performed, amongst other physiological endpoints.

## Discussion

### Theoretical advantages of perfused human organs

*Ex vivo *human organs allow a three dimensional biological system with a certain degree of retained physiological functions, native cellular architecture, and extracellular matrix that are superior to laboratory cell- and tissue-based bioassays. Toxicity of drugs can be observed directly on the *ex vivo *organ using interval biopsy and other physiological sampling. This should, in theory, be comparable, if not equivalent, to phase 1/2 studies for drug testing. This setup also allows for the study of a trial drug on one single organ, eliminating possible interference from other physiological systems. Overall, use of *ex vivo *human organs in drug trials can generate useful human data in order to fast-track a trial drug for more advanced clinical studies. Having said this, the use of perfused human organ research carries conceptual, practical, and ethical limitations.

### Limitations of perfused human organs

#### 1. Ex vivo *it is!*

Once extracted from the human body, an organ is instantly cut off from the original physiological milieu in terms of blood supply, nervous modulation, immuno-regulation, and thermo-homeostasis. We can use matched whole blood at core body temperature and simulate the flow pattern due to normal heart beat and blood pressure, but we cannot reproduce those dynamic regulatory changes due to nervous and immune regulation. Even if we use matched whole blood, how can we simulate the variation in vasoconstriction/vasodilatation, blood sugar, amino acids, and lipids, which normally happens at least three times a day after meals? Can we say these issues are irrelevant to the profile and toxicity of the drug tested? If the perfused human organ functions with a different set of physiological variables, how can we distinguish it from a scaled-up tissue model in the guise of an organ?

#### 2. An organ in isolation

In real life, drug effects and metabolisms affect more than one organ. One example would be the liver exerting a first pass effect, followed by the normal physiological pattern of the kidneys excreting a major proportion of the drug. Not to overlook how the binding of the drug is affected by the serum albumin level (which may vary physiologically, but is not reproducible ex vivo), and the total adipose tissue, which affects the ultimate distribution volume of the drug (again, not reproducible ex vivo). Therefore, a perfused *ex vivo *organ in isolation is not comparable to its natural *in vivo *counterpart when a global (hence physiologically closer to real human) picture for the tested drug is necessary. It is like taking a snapshot from one angle only, and then trying to work out the entire panorama. How far can the physiological extrapolation realistically be projected?

#### 3. Cost-effectiveness

Curtis *et al*[4] stated in their article that the Ex Vivo Metrics^© ^system is not a high throughout system, but did not detail either the overhead or running costs. Nor did they mention the criteria of usage termination for the organ (i.e., what level of toxicity can the perfused organ suffer before it is deemed unusable?). This raises the question of overall cost-effectiveness, which is an agenda item in N2B translational medicine.

#### 4. Ethics

Fresh human organs are usually scarce and have to be allocated between two competing groups: the bench and the bedside. Team leaders in drug development can justify the need for a perfused liver to expedite a drug trial that theoretically can help millions of people; equally, that same organ can dramatically extend the life of a person who is dying of fulminant liver failure. How do we draw the line and who should do it? Here, translational medical professionals can step in to act as the arbitrator in assessing the projected and realistic needs from differing parties; and finally, to facilitate a decision that best meets the demands from all sides. (Hence, a bench-to-bedside "N2B" question with a bedside-to-bench "B2N" feedback loop in decision making).

## Conclusion

The importance of organs as the building blocks for normal functioning of the human body has long been ingrained in the history of western medicine. It is perfectly logical to follow this line of thought: to extract a human organ for studying the effects of novel drugs in order to be one step further along than cell- or tissue-bioassays. However, we must not forget that our human body is an interactive complex of multiple systems governed by feedback loops, and human organs are mere anatomical landmarks in the living process. With our present scientific technologies, extracting and revitalizing one organ *ex vivo *is still no match for the same organ functioning naturally *in vivo*. Having considered the limitations inherent to data procured from such *ex vivo *models, we must also balance the cost-effectiveness of using such models for pre-clinical drug screening within the domain of prevailing research ethics and various regulatory bodies. As a newcomer in translational medicine, I am indeed concerned about just how far this technology of *ex vivo *human organ research would lead us to; I sincerely hope it would not be as dramatic or tragic as depicted by Mary Shelley in her novel, "Frankenstein"[5].

## Competing interests

The author declares that they have no competing interests.

## References

[B1] Pardridge WM (2003). Translational science: what is it and why is it so important?. Drug Discov Today.

[B2] Ott MG, Schmidt M, Schwarzwaelder K, Stein S, Siler U, Koehl U (2006). Correction of X-linked Chronic Granulomatous Disease by Gene Therapy, Augmented by Insertional Activation of MDS1-EVI1, PRDM16 or SETBP1. Nat Med.

[B3] Palfi S (2008). Towards gene therapy for Parkinson's disease. The Lancet Neurology.

[B4] Curtis CG, Bilyard K, Stephenson H (2008). Ex Vivo Metrics, a preclinical tool in new drug development. J Transl Med.

[B5] Davies H (2004). Can Mary Shelley's Frankenstein be read as an early research ethics text?. Med Humanit.

